# Comparison of Efficiencies of Michigan Neuropathy Screening Instrument, Neurothesiometer, and Electromyography for Diagnosis of Diabetic Neuropathy

**DOI:** 10.1155/2013/821745

**Published:** 2013-05-22

**Authors:** Turkan Mete, Yusuf Aydin, Mustafa Saka, Halise Cinar Yavuz, Sule Bilen, Yavuz Yalcin, Berna Arli, Dilek Berker, Serdar Guler

**Affiliations:** ^1^Ankara Numune Education and Research Hospital, Department of Endocrinology, Sıhhiye, 06622 Ankara, Turkey; ^2^Duzce University, Faculty of Medicine, Endocrinology and Metabolism Department, Turkey; ^3^Ankara Numune Research and Education Hospital, Department of Neurology, Turkey

## Abstract

*Aim*. This study compares the effectiveness of Michigan Neuropathy Screening Instrument (MNSI), neurothesiometer, and electromyography (EMG) in detecting diabetic peripheral neuropathy in patients with diabetes type 2. *Materials and Methods*. 106 patients with diabetes type 2 treated at the outpatient clinic of Ankara Numune Education and Research Hospital Department of Endocrinology between September 2008 and May 2009 were included in this study. Patients were evaluated by glycemic regulation tests, MNSI (questionnaire and physical examination), EMG (for detecting sensorial and motor defects in right median, ulnar, posterior tibial, and bilateral sural nerves), and neurothesiometer (for detecting alterations in cold and warm sensations as well as vibratory sensations). *Results*. According to the MNSI score, there was diabetic peripheral neuropathy in 34 (32.1%) patients (score ≥2.5). However, when the patients were evaluated by EMG and neurothesiometer, neurological impairments were detected in 49 (46.2%) and 79 (74.5%) patients, respectively. *Conclusion*. According to our findings, questionnaires and physical examination often present lower diabetic peripheral neuropathy prevalence. Hence, we recommend that in the evaluation of diabetic patients neurological tests should be used for more accurate results and thus early treatment options to prevent neuropathic complications.

## 1. Introduction

Diabetic neuropathy is the most common microvascular complication of diabetes, and it is a major cause of morbidity and mortality. Neuropathy is estimated to be present in 10%–90% of the patients with diabetes although it changes according to diagnostic criteria and patient population. Diabetic peripheral neuropathy is the most common type of diabetic neuropathy, and it is frequently used synonymously with it [1]. 

Early diagnosis and appropriate treatment are important to prevent disease complications, especially diabetic foot and ulceration, but there is not a single and simple method that can be used to diagnose diabetic peripheral neuropathy. According to American Diabetes Association (ADA) recommendations, diabetic peripheral neuropathy diagnosis in clinical practice is made in the presence of signs and symptoms of peripheral nervous system dysfunction after other causes of neuropathy are excluded in patients with diabetes. Considering that 50% of the patients with diabetic peripheral neuropathy have no symptoms consistent with neuropathy, neurological examination of the patients should be carefully performed. To confirm the diagnosis, quantitative electrophysiological tests and sensory and autonomic function tests can be performed [2, 3]. 

The aim of this study was to compare the effectiveness of Michigan Neuropathy Screening Instrument (MNSI), neurothesiometer, and electromyography (EMG) in detecting diabetic peripheral neuropathy in patients with diabetes type 2.

## 2. Patients and Methods

### 2.1. Patients

The study was conducted in Ankara Numune Education and Research Hospital Department of Endocrinology from September 2009 to February 2010. 106 type 2 diabetes patients with or without symptoms of neuropathy were enrolled in the study. 

Patients who had conditions that could present with neuropathy such as hereditary sensory neuropathy, vitamin B12 or folate deficiency, paraneoplastic conditions, autoimmune diseases, uremia, hypothyroidism, and ethanol abuse were excluded. 

Patients' height and weight were measured and used to calculate body mass index (BMI). Blood pressure was recorded in supine position after 5 minutes of rest using oscillometry. Hypertension was defined as ≥140/90 mmHg at examination or presence of antihypertensive treatment. 

Medication history (use of insulin, oral antidiabetic, antihypertensive, and lipid-lowering drugs) was noted. Retina evaluation was performed by an ophthalmologist. Retinopathy was classified into five categories using an international system of classification: absence of retinopathy, mild nonproliferative retinopathy, moderate nonproliferative retinopathy, severe nonproliferative retinopathy, and proliferative retinopathy [4].

The study complied with the declaration of Helsinki and was approved by the local research ethics committee. All the subjects gave written informed consent.

### 2.2. Laboratory Examinations

Blood samples were obtained at 8 a.m. after 12 hours of fasting. Lipid profile (total cholesterol (TC), triglycerides (TG), and high-density lipoprotein cholesterol (HDL-C)) were measured with colorimetric enzymatic method (Aerost device, Abbott Diagnostics, USA). Low-density lipoprotein cholesterol (LDL-C) was calculated by Friedewald's formula. Hyperlipidemia was considered present when lipid-lowering drugs were in use or when samples at admission showed total cholesterol ≥200 mg/dL or triglycerides ≥150 mg/dL. Glycosylated hemoglobin (HbA1c) was measured by immunoturbidimetric method (C8000 device, Abbott Company, USA). For the diagnosis of nephropathy, the patients were asked to collect urine for 24 hours after urinating the first urine in the morning and not to do any exercise 24 hours before and during the collecting procedure. Urinary albumin excretion (UAE) was measured with Multigent microalbumin (*μ*Alb) turbidimetric immunoassay method (Aerost device, Abbott Diagnostics, USA). We considered <30 mg/day as normoalbuminuria, 30–300 mg/day as microalbuminuria, and >300 mg/day as macroalbuminuria. Microalbuminuria is defined as a total of three positive 24-hour urine collections measured at different days to confirm the diagnosis. Plasma creatinine levels of the patients were measured, and glomerular filtration rate (GFR) was calculated using the Chronic Kidney Disease Epidemiology Collaboration (CKD-EPI) formula [5]. B12 levels of all patients were measured by chemiluminescence method on an ADVİA Centaur XP analyser.

### 2.3. Assessment of Diabetic Neuropathy

All patients were evaluated for diabetic peripheral neuropathy using MNSI, EMG, and neurothesiometer. All tests for neurological assessment were performed on the same day.

#### 2.3.1. MNSI

A 15-item questionnaire form of MNSI consisting of yes/no questions was applied to all the patients. 13 items assess symptoms of diabetic peripheral neuropathy, 1 item assesses peripheral vascular disease, and 1 item assesses general asthenia [6]. 

*Michigan Neuropathy Screening Instrument*. Answer the following yes or no questions based on how you feel in your legs and feet.

1. Are your legs and/or feet numb?
2. Have you ever had burning sensation in your legs and/or feet?
3. Are your feet too sensitive to touch?
4. Do you get muscle cramps in your legs and/or feet?
5. Have you ever had any prickling feelings in your legs or feet?
6. Does it hurt when the bed covers touch your skin?
7. When you get into the tub or shower, are you able to distinguish the hot water from the cold water?
8. Have you ever had an open sore on your foot?
9. Has your doctor ever told you that you have diabetic neuropathy?
10. Do you feel weak all over most of the time?
11. Are your symptoms worse at night?
12. Do your legs hurt when you walk?
13. Are you able to sense your feet when you walk?
14. Is the skin on your feet so dry that it cracks open?
15. Have you ever had an amputation?

Source: a practical two-step quantitative clinical and electrophysiological assessment for the diagnosis and staging of diabetic neuropathy. Diabetes Care 1994; 17: 1281-9.

After the questionnaires, patients were evaluated neurologically.

- In physical examination, feet were evaluated for deformity, dry skin, callus, infection, and ulceration. Foot deformities included prominent metatarsal heads, hallux valgus, joint subluxation, and Charcot joint. One point was given if any of these signs were present and an additional one point was given if ulceration was present.
- Vibration sense was evaluated using 128 Hz vibration fork. Vibrating fork was located on the interphalangeal joint of the right great toe. If the patient could not perceive vibration, two points were given. If the patient perceived vibration on the great toe, diaposone was located over ankle (inner malleolus) while it was still vibrating and the patient was asked to compare vibrations from two locations. If vibration was perceived better in ankle, 1 point was given. If no difference could be found, no point was given. Zero point was accepted as normal, 1 point showed mild-moderate deficit, and 2 points showed a severe deficit.
- Achilles reflex was observed and reported as absent, decreased, or normal. Patients with normal Achilles reflex were given 0 point while patients with decreased Achilles reflex got 0.5 point and patients with no reflex got 1 point.

Positive responses and abnormal physical examination findings were recorded in the questionnaire form. In the questionnaire form risk of neuropathy was accepted to increase with higher number of positive responses. Diabetic peripheral neuropathy was diagnosed in patients with a physical examination score ≥2.5. In this study MNSI was accepted as a diagnostic test according to ADA recommendations.

#### 2.3.2. Neurothesiometer

For neurothesiometer evaluation a TSA II device (Neurosensory Analyzer Model TSA II, Medoc Ltd., Israel) was used. TSA II is a device used to quantitatively evaluate thin fiber dysfunction. Thermal tests quantitatively measure hot, cold sensations and pain sensation induced by these and compare them with corresponding age group. Deviance from normal values may show presence of peripheral nervous system disease. In vibratory tests, the same type measures are made and compared with vibration thresholds of the population. 

Patients sat comfortably in a quiet room with a room temperature of 18–22°C. Before the test, the patient was informed about the procedure and a trial was made without recording. Thermod was fixed to the surface to be tested (in our study to right palmar thenar and to right foot 1. metatarsal regions), with metal surface contacting adequately with skin. For sensory measurements, patients were asked to push the button as soon as they perceived the heat changes. In thermal test mode, thermod was attached to patients' skin (first to the right palmar thenar surface, then to the right foot plantar 1. metatarsal region). Thermod included semiconductors that form temperature gradient between upper and lower stimulating surfaces. Test was started using 30–32°C adaptation temperature. After a few seconds patients could not perceive any difference in temperature. For threshold detection, a perceivable heat stimulus was produced by the device. Data were recorded by a computer when the patient pushed the button in her/his hand and each cyclus of the measurement was completed after this. After the measurement, thermod was returned to adaptation temperature. A waiting period of a few seconds was allowed before the second stimulus. 

TSA II measured threshold values for 4 sensory modalities.

- Heat sensation conducted with C fibers was generally 1-2°C above adaptation temperature.
- Cold sensation perceived by A delta fibers was generally 1-2°C below adaptation temperature.
- The threshold value of pain sensation induced by heat which is generally conducted with C fibers and partially A delta fibers was approximately 45°C.
- The pain sensation induced by cold which is conducted both with C and A delta fibers had a threshold value of 10°C. For pain measurements patients were told that it was not a pain tolerance test and they should push the button as soon as they perceive pain.
- Sense of vibration was measured from plantar side of the right foot, the 1st metatarsal region. Stimulus threshold values for vibration sense were 0.1–130 microns/s. Two types of stimuli were used in this method. One of them was increasing stimulus intention till it was perceived and the other was decreasing stimulus intention till it could not be perceived. Then printouts were obtained.

Threshold values for heat, cold, and vibration were measured in all the patients. Values were compared with the same age normal population using TSA II software [7]. 

#### 2.3.3. EMG

EMG was performed in all the patients involved in the study. Nerve conductions were studied using Nihon Kohden MEB-9104K neuropack *μ* device (Tokyo, Japan). Nerve conduction studies were as follows: ulnar nerve sensory and motor conductions in upper extremity and deep peroneal motor, posterior tibial motor, and sural nerve conductions in both lower extremities. Based on the results of a study of the Turkish population, the normal limits for nerve conduction evaluations were determined as follows: distal latency for ulnar nerve motor conduction was 3.3 ms, Compound Muscle Action Potential (CMAP) amplitude was 7 mV, motor conduction velocity was 39.6 m/s; ulnar nerve distal sensory conduction speed was 37.3 m/s, amplitude was 7 *μ*V; deep peroneal nerve motor conduction distal latency was 5.8 ms, amplitude was 3.6 mV, conduction speed was 40.9 m/s, F response latency was 52 ms; sural nerve conduction speed was 33.8 m/s, amplitude was 5 *μ*V. At least two pathological nerve conductions, one of which was in the sural nerve, led to symmetric polyneuropathy diagnosis [8]. 

### 2.4. Statistical Analyses

Statistical evaluations of the results of this study were done using IBM SPSS (Statistical Package for Social Sciences) for Windows 20.0. In the assessment, in addition to descriptive statistical methods (frequency, mean, and standard deviation), *t*-test was used for the comparison of data and logistic regression analysis was used for diagnostic comparisons. We used a 95% confidence interval and considered a *P* value <0.05 as statistically significant. Sensitivity and specificity of diagnostic tests were calculated according to the gold standard.

## 3. Results

Demographic data of 106 patients with diabetes type 2 included in this study are given in Table 1. Mean age of the patients was 49.55 ± 10.28 years. Forty-three patients were male. Mean duration of diabetes was 99.48 ± 80.96 months (0–312 months). Four patients recently had diabetes diagnosis and they were not receiving diabetes treatment at the time of testing. Fifty-seven patients were using oral antidiabetics, 24 were using insulin, and 21 were using insulin and oral antidiabetic combination. 46 patients had hypertension and 41 of them were on antihypertensive treatment. Of the patients with hyperlipidemia, 21 patients were on statins, and 14 were on fenofibrate treatment. Mean values for LDL cholesterol and triglyceride levels were 108.1 mg/dL and 176.4 mg/dL, respectively. Mean BMI was 30.3 kg/m^2^. 52 patients were obese. All patients had normal serum vitamin B12 levels.

Mean HgbA1c value of the patients was 8.4 ± 2.3 (5.3–15.9). Retinopathy was detected in 26 patients (16 mild nonproliferative, 4 severe nonproliferative, and 6 proliferative retinopathy) and nephropathy was detected in 28 patients (23 patients had microalbuminuria and 5 had macroalbuminuria). Mean glomerular filtration rate was 92 mL/min/1.73 m^2^.

In the assessment for neuropathy, the mean score of the patients obtained in the MNSI questionnaire form was 6.7 ± 2.7 (maximum 12, minimum 3 points). After the questionnaire, physical examination part of MNSI was applied to the patients. According to MNSI, diabetic peripheral neuropathy (score ≥2.5) was detected in 34 patients (32.1%). Mean diabetic period for the 34 patients diagnosed with diabetic peripheral neuropathy by MNSI was 125.9 (0–300) months. The diabetic period was longer compared to the patients not diagnosed by MNSI, and the difference was statistically significant (*P* = 0.04). Mean HbA1c level was 8.6% (5.8–15.9). The difference between the groups was not statistically significant (*P* = 0.63). While 13 patients had hypertension, 25 patients were diagnosed with hyperlipidemia and 11 patients were on lipid-lowering treatment because of this. 16 patients had obesity. There was no statistically significant difference between the groups in terms of hypertension, hyperlipidemia, and obesity (*P* = 0.72, *P* = 0.07, and *P* = 0.08, resp.). Retina examination showed proliferative retinopathy in 6 patients, mild nonproliferative retinopathy in 4 patients, and severe nonproliferative retinopathy in 3 patients. Retinopathy was higher in the group diagnosed with diabetic peripheral neuropathy by MNSI compared to the group without diabetic neuropathy (*P* = 0.01). In the neuropathic group, 12 patients had microalbuminuria and 3 had macroalbuminuria. There was no significant difference between the groups (*P* = 0.089, *P* = 0.18, resp.).

As a result of EMG evaluations, neuropathy was diagnosed in 54 patients (50.9%). 49 patients had diabetic polyneuropathy (46.2%) and 5 had mononeuropathy. Of these patients, 10 patients had sensory neuropathy, 9 patients had motor neuropathy, and 30 patients had both sensory and motor neuropathies. 

 Neurothesiometer evaluations revealed change in heat and/or vibration thresholds in 79 of 106 patients (74.5%). Increase in threshold was detected in cold sensation in 5 patients and in heat sensation in 10 patients. Eighteen patients had threshold increase both in cold and heat sensations, 13 patients had increase in vibration sense threshold, and 33 patients had increase in both thermal (cold and heat) and vibration sense thresholds. 

When only MNSI score was used for diagnosis, diabetic peripheral neuropathy was detected in 34 of 106 patients (32.1%). Polyneuropathy findings were detected in 49 patients (46.2%) with EMG and in 79 patients (74.5%) with neurothesiometer (Table 2).

30 (91.2%) of the patients diagnosed with diabetic peripheral neuropathy by MNSI were diagnosed with thin fiber neuropathy by neurothesiometer and 20 (58.8%) had EMG consistent with diabetic peripheral neuropathy. In neurothesiometer evaluation, threshold increases were detected in both thermal and vibration senses in 12 patients, in thermal sense in 4 patients, in heat and vibration senses in 7 patients, only in vibration sense in 4 patients, in cold and vibration senses in 1 patient, and in heat sense in 2 patients. Neurothesiometer evaluation was normal in 4 patients. In EMG evaluation 14 patients had distal peripheral sensory and motor neuropathy, 4 patients had only motor, and 2 patients had only sensory neuropathy. EMG was normal in 14 patients who had neuropathy detected by MNSI. 

 In neurothesiometer evaluation increase in thermal and/or vibration senses consistent with diabetic peripheral neuropathy was detected in 79 patients included in this study. In 30 (39.2%) of these patients neuropathy was also detected by MNSI and in 45 (42%) patients polyneuropathy was detected by EMG. MNSI questionnaire form revealed findings consistent with neuropathic pain (number of positive responses ≥7) in 44 patients who were detected to have diabetic peripheral neuropathy by neurothesiometer. These results showed that probability of neuropathy according to both MNSI questionnaire form and MNSI physical examination results was high in patients detected to have nerve conduction defects by neurothesiometer (*P* < 0.01). In 17 (37.5%) patients detected to have dysfunction by neurothesiometer, neuropathy could not be detected by MNSI or EMG. 

In EMG evaluation 49 patients (46.2%) had diabetic polyneuropathy. In 20 (37%) of these patients, neuropathy was also present according to MNSI examination score. In patients detected to have nerve conduction deficit with EMG, probability of being symptomatic (questionnaire score ≥7) according to the questionnaire form was 58.5%. No significant difference could be detected between EMG and MNSI (*P* > 0.05). In neurothesiometer evaluation nerve conduction deficit was detected in 89.7% of the patients who had dysfunction consistent with neuropathy in EMG (*P* < 0.001). In 4 patients (11.8%) who had dysfunction in EMG both MNSI and neurothesiometer evaluations were normal. 

EMG method had a sensitivity of 55% and specificity of 58% based on MNSI. Positive and negative predictive values were 38% and 73%, respectively. Neurothesiometer method had a sensitivity of 91% and specificity of 50% based on MNSI. Positive and negative predictive values were 39% and 88%, respectively. EMG and neurothesiometer together had a sensitivity of 53% and specificity of 62%. Positive and negative predictive values when two methods used together were 40% and 73%, respectively. 

## 4. Discussion

 Distal symmetric polyneuropathy is the most common form of diabetic neuropathy. Epidemiologic studies have identified the duration and severity of hyperglycemia as major risk factors for the development of diabetic neuropathy in patients with diabetes [9, 10]. In our study we identified a longer diabetic period in the group diagnosed with diabetic peripheral neuropathy than in the group without diabetic neuropathy. However, we did not find any difference in the glycemic regulations of the groups. A study on the role of glucose control on the neuropathy in patients with type 1 and type 2 diabetes suggests that in type 1 diabetes, glucose control has a large effect on the prevention of neuropathy; therefore, future efforts should continue to concentrate on this avenue of treatment. In contrast, patients with type 2 diabetes, glucose control does not play a significant role in the prevention of neuropathy [11]. Vascular risk factors also appear to be associated with the risk of developing diabetic neuropathy. Evidence of this association comes from the European Diabetes (EURODIAB) Prospective Complications Study [12]. In addition to duration of diabetes and glycosylated hemoglobin value, the incidence of neuropathy was significantly associated with increased triglyceride level, body mass index, smoking, and the presence of hypertension at baseline. Several of these risk factors are markers of insulin resistance. Similarly, in our study, we evaluated 106 patients with diabetes type 2 not only in terms of neuropathy prevalence but also for vascular risk factors. We found no significant difference between the 34 patients diagnosed with diabetic peripheral neuropathy by MNSI and those without a diagnosis of neuropathy in terms of hypertension, hyperlipidemia, and obesity prevalence. However, we observed an increased rate of retinopathy, another microvascular complication of diabetes, in patients diagnosed with neuropathy. 

 In this study we compared the effectiveness of three different methods that can be used in the diagnosis of diabetic polyneuropathy. MNSI was used as a diagnostic method based on symptoms and signs. EMG and neurothesiometer were used to confirm diagnosis and to evaluate the diagnostic efficiency of each of the methods used in this study. 

MNSI score was ≥2.5 in 34 patients involved in our study and diagnosis of diabetic neuropathy was made. This test was developed by Neurology Department of Michigan University and had first been applied to 56 patients with diabetes. These patients had also been evaluated for diabetic neuropathy according to San Antonio Consensus Statement and Mayo Clinic protocol. Neuropathy had been detected in 28 of 29 patients that had an MNSI score >2. In questionnaire form of MNSI, 20 patients with diabetic neuropathy and 18 patients without diabetic neuropathy had given positive responses to ≤6 questions. But 2 patients that had not had neuropathy and 14 patients that had neuropathy had given positive responses to ≥7 questions. These results had shown that many patients without neuropathy gave positive responses to ≤6 questions [6]. Similarly, in our study of 34 patients who were diagnosed as diabetic neuropathy based on MNSI, 24 gave positive responses to ≥7 questions, and of the 72 patients who were not diagnosed as diabetic neuropathy, 28 gave positive responses to ≥7 questions. These results suggest that diagnosing neuropathy depending only on symptoms can be misleading. 

In the second part of our study, EMG was performed to all the patients included in our study. Like our study nerve conduction studies were made in the second part of the test recommended by Michigan University to confirm the diagnosis and to grade neuropathy. In the section called Michigan Diabetic Neuropathy Score (MDNS), patients were evaluated with neurological examination, nerve conduction studies, Neuropathic Deficiency Score (NDS), vibration threshold, autonomic function tests, and Neuropathy Symptom Profile (NSP), and results were graded from 0 to 3. Abnormal nerve conduction results were seen in 69% of patients detected to have neuropathy with MNSI [6]. 

In our study, in 20 of the patients (58.8%) diagnosed with neuropathy by MNSI, nerve conduction defect was detected with EMG. In our study, apart from evaluation made by Michigan University, EMG was also applied to patients who could not be diagnosed with MNSI. In this group of patients MNSI failed to diagnose neuropathy, and peripheral nerve conduction deficits were observed in 29 patients. Although abnormal electrodiagnostic tests are not considered as diagnostic by themselves, this result has shown that nerve conduction deficits developed at a high rate even in patients who were not diagnosed with neuropathic based on signs and symptoms. These patients have an increased risk for complications based on neuropathy and they should be followedup. 

In this multicenter cross-sectional study done in Turkey, neurologic examinations and nerve conduction studies along with clinical diabetic neuropathy score and Leeds Assessment of Neuropathic Symptoms and Signs pain scale were performed on 1113 patients with diabetes to determine the prevalence of diabetic peripheral neuropathy. Prevalence of diabetic peripheral neuropathy determined only by clinical examination was 40.4% and it rose to 62.2%, when nerve conduction studies were combined with clinical examination [13]. Similarly, in our study, we observed that using clinical examinations and nerve conduction studies together is important for accurate diagnosis of DPN.

If neuropathy mainly affects thin, unmyelinated nerve fibers, electrophysiological tests are frequently normal. Neurothesiometer device was developed in recent years for quantitative sensory testing (QST) (CASE IV device was developed by Peter Dyck and colleagues). It allows appropriate evaluation of threshold values for vibration, heat, and pain senses. Neurothesiometer allows evaluations in which intensity and features of the stimulus are controlled well (e.g., tests applied to the same patient at different times and in different centers give the same results). QST is a valuable device to follow progression of neuropathy in patients with diabetic neuropathy [14]. 

In our study, neurothesiometer evaluation was applied to all the patients as a QST and threshold values for vibration, heat, and pain were evaluated. In neurothesiometer evaluation increased threshold was detected in 79 patients. In 30 (39.2%) of these patients neuropathy was present in MNSI. According to MNSI questionnaire form positive response number was ≥7 in 44 (57.7%) patients. In 30 of 34 patients diagnosed with neuropathy by MNSI, neurothesiometer showed nerve conduction deficit. In 89.7% of patients detected to have a dysfunction consistent with neuropathy in EMG, nerve conduction deficit was also detected with neurothesiometer (*P* < 0.001). 

This test is a noninvasive screening test, but it cannot be diagnostic by itself because of its subjective nature. It was used to evaluate neuropathy incidence in 1011 patients who had a diagnosis of diabetes for more than 10 years in Spain to evaluate neuropathy prevalence. Diagnosis of neuropathy was confirmed by DN4 questionnaire form developed by French Neuropathic Pain Group which includes both history and physical examinations. After the study neuropathy was detected in 39.6% and subclinic neuropathy was detected in 36.8% of the patients. This study suggests that polyneuropathy is underdiagnosed and quantitative evaluation will be helpful [15].

In a study by Kincaid et al., clinical evaluation, nerve conduction study, and neurothesiometer were compared in the diagnosis of diabetic neuropathy. In a multicenter study involving 227 patients with diabetes mellitus, vibration threshold was measured with neurothesiometer, and peroneal, tibial, and sural nerves were evaluated with nerve conduction studies. Results of this study showed that these tests cannot be used interchangeably, but they could be complementary [16].

A study of 152 patients with diabetic peripheral neuropathy gave the patients electrodiagnostic evaluation and quantitative vibration perception thresholds testing with the Vibratron II and neurothesiometer and concluded that vibration perception thresholds determined with the neurothesiometer are less variable than the thresholds determined with the vibratron and they are more reflective of peripheral nerve function. The results of this study indicate that the neurothesiometer can be used reliably in clinical research trials [17].

In a study with 2022 diabetic patients, peripheral polyneuropathy was diagnosed by vibration perception threshold at the tip of both great toes using a 128-Hz tuning fork and a neurothesiometer. Vibration perception threshold was also measured in 175 nondiabetic control subjects to define normal values. Finally, the vibration perception threshold measured by the neurothesiometer was 2.5 times higher in patients with an abnormal tuning fork test. The plot of the difference of both methods against their mean yielded a good agreement of the two VPT measurements [18].

## 5. Conclusions

In conclusion, this study evaluated 106 diabetic patients and found dysfunction consistent with neuropathy in 34 patients with MNSI, in 54 patients with EMG, and in 79 patients with neurothesiometer. Although electrodiagnostic evaluations do not have diagnostic value themselves according to ADA, they are thought to confirm the diagnoses. However in our study a higher rate of neuropathy was observed in electrophysiologic tests than in anamnesis and physical examination. Symptoms consistent with neuropathy were also detected in patients who were diagnosed with neuropathy by EMG and neurothesiometer but were not diagnosed neuropathy by MNSI. According to these data, diagnosing with diabetic neuropathy based only on anamnesis and physical examination will cause underdiagnosis of the problem. 

MNSI can be performed easily by a physician at office conditions although EMG requires an experienced neurologist and neurology laboratory. To perform neurothesiometer, help is needed from trained staff. But if early diagnosis of diabetic peripheral neuropathy and thus prevention of complications with high morbidity are taken into account, common use of electrodiagnostic methods can be considered to be cost-effective. 

## Figures and Tables

**Table 1:** General characteristics of the study group.

	Total
Number of cases	106
Age (year)*	49.55 ± 10.28
Sex: male/female	43/63
BMI (kg/m^2^)	30.3
DM disease length* (months)	99.48 ± 80.96
Recently diagnosed DM, *N* (%)	4 (3.77)
Not taking a treatment, *N* (%)	4 (3.8)
Taking treatment, *N* (%)	102 (96.22)
Oral antidiabetics	57 (55.88)
Insulin	24 (23.53)
Oral antidiabetics + insulin	21 (20.58)

DM: diabetes mellitus; *mean ± standard deviation.

**Table 2:** Results of MNSI, EMG, and neurothesiometer.

	Total (*n*: 106)
MNSI questionnaire score*	6.7 ± 2.7
Mean examination score*	1.55 ± 0.75
Diabetic peripheral neuropathy with MNSI (MNSI ≥ 2.5), *N* (%)	34 (32.1)
Diabetic peripheral neuropathy with EMG *N* (%)	49 (46.2)
Diabetic peripheral neuropathy with neurothesiometer *N* (%)	79 (74.5)

*Mean ± standard deviation.
